# Integrating Structural Characterization, Network Pharmacology, and Experimental Validation Reveals the Anti‐Liver Aging Mechanism of *Acorus tatarinowii* Polysaccharides

**DOI:** 10.1002/cbdv.71815

**Published:** 2026-10-12

**Authors:** Yikuan Du, Jiahui Li, Yuting Lin, Jiawen Luo, Weien Zhang, Feng Chen, Zhonghan Liang, Jiaxin Zhu, Jiaqi Li, Chenyue Yuan, Yidian Liu, Xuan Liu, Yang Zhang, Qiaohong Zhong, Yanling Jiang, Songsong Yang, Chunling Ma, Zhi Chen, Chun Yang

**Affiliations:** ^1^ Dongguan Key Laboratory of Translational Medicine and Innovative Drugs The Tenth Affiliated Hospital of Southern Medical University Dongguan China; ^2^ Dongguan Key Laboratory of Stem Cell and Regenerative Tissue Engineering The First Dongguan Affiliated Hospital School of Basic Medical Sciences Guangdong Medical University Dongguan China; ^3^ The First Dongguan Affiliated Hospital Guangdong Medical University Dongguan China

**Keywords:** *Acorus tatarinowii* polysaccharides, liver aging, network pharmacology, oxidative stress, inflammatory, structural characterization

## Abstract

Liver aging is critical in chronic liver disease progression and systemic aging. Certain polysaccharides from traditional Chinese medicine have attracted increasing interest for their potential to delay liver aging. *Acorus tatarinowii* exhibits significant hepatoprotection, and its polysaccharides are key bioactive components against aging‐related inflammation. However, the mechanism by which *Acorus tatarinowii* polysaccharides (ATP) protect against liver aging remains poorly understood. The structural features of ATP were characterized, and network pharmacology and bioinformatics were employed to predict key targets and pathways. Furthermore, the hepatoprotective effects of ATP were evaluated in D‐galactose‐induced aging rats. ATP (56.1 kDa) consisted of rhamnose, arabinose, galactose, glucose, xylose, and galacturonic acid. 272 potential targets were identified. Functional enrichment suggested that these targets were mainly involved in inflammatory response, oxidative stress, Toll‐like receptor signaling, and the Keap1–Nrf2 pathway. WGCNA and machine learning identified TLR4 and Nrf2 as potential key targets. In vivo experiments showed that ATP significantly alleviated hepatic atrophy and steatosis, suppressed hepatic TLR4, NF‐κB, and HO‐1 expression, and activated Nrf2 expression. These findings suggest that ATP ameliorates liver aging, and its hepatoprotective effect is correlated with the regulation of the TLR4/NF‐κB and Nrf2 pathways, indicating its potential as a natural anti‐aging agent for liver protection.

## Graphical abstract

ATP is extracted and structurally characterized. Network pharmacology, WGCNA and machine learning identify TLR4 and Nrf2 as core targets. In D‐gal‐induced aging rats, ATP relieves hepatic atrophy and steatosis, suppresses hepatic TLR4, NF‐κB, and HO‐1 expression, and up‑regulates Nrf2 expression, showing its promising potential for counteracting hepatic senescence. Note: This graphical abstract was originally drawn by the authors using Adobe Illustrator.

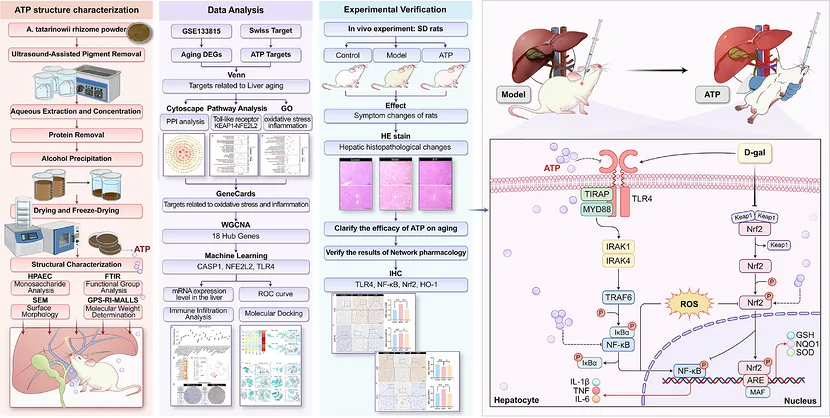

## 1 Introduction

Recent studies, including those in *Nature*, highlight that the liver, as the central hub for metabolism and detoxification, exhibits pronounced age‐associated structural and functional alterations that disproportionately impact systemic homeostasis [1, 2, 3]. With advancing age, the liver undergoes a series of structural and functional alterations, including reduced blood flow, diminished hepatocyte regenerative capacity, increased immune inflammation and fibrotic deposition, as well as a decline in metabolic competence [4]. Liver aging not only exacerbates metabolic disorders and metabolic syndrome [5], but also increases the risk of developing diseases such as fatty liver, cirrhosis, and liver cancer [6]. Meanwhile, liver aging propagates systemic aging to distal organs such as the kidney, brain, and lungs through the secretion of senescence‐associated secretory phenotype (SASP) factors, including transforming growth factor beta (TGFβ), leading to multi‐organ dysfunction and posing a severe threat to human health and longevity [7]. For delaying liver aging, the primary therapeutic approaches currently involve broad‐spectrum anti‐aging drugs such as senolytics, senomorphics, and nicotinamide adenine dinucleotide (NAD^+^) precursors. However, these therapeutic approaches are limited in clinical application due to issues such as severe adverse effects, low bioavailability, and potential hepatotoxicity [8]. Therefore, the search for safe and effective anti‐liver‐aging treatments derived from natural products has become a research hotspot.

In recent years, polysaccharides derived from traditional Chinese medicine have attracted considerable attention owing to their wide range of sources, low toxicity, minimal side effects [9], and strong support from national policies [10] A growing body of evidence indicates that polysaccharides, characterized by their structural diversity, possess multiple pharmacological activities, including immunomodulatory, antioxidant, gut microbiota–modulating, and anti‐inflammatory effects, thereby showing great potential in the prevention and treatment of liver aging [11, 12]. For example, Astragalus polysaccharides regulate the AMP‐activated protein kinase/mechanistic target of rapamycin (AMPK/mTOR) signaling pathway to promote mitochondrial autophagy, alleviate oxidative stress, and delay hepatocyte senescence [13]; *Arctium lappa L*. polysaccharides alleviate oxidative stress and inflammatory responses in the aging liver of mice by modulating intestinal homeostasis [14]. However, although natural polysaccharides have shown great potential in combating liver aging, rigorous scientific evidence substantiating their anti‐liver‐aging efficacy remains insufficient. On the one hand, some existing studies still primarily rely on assays of antioxidant enzyme activity to evaluate the anti‐liver‐aging or hepatoprotective effects of polysaccharides [15, 16, 17], such as superoxide dismutase, while further exploration of upstream signaling pathways has been relatively limited. On the other hand, although some studies have focused on upstream mechanisms [18, 19], there is a lack of systematic elucidation of the molecular details regarding how polysaccharides specifically regulate these pathways [20]. Therefore, identifying traditional Chinese medicine polysaccharides with hepatic aging‐protective activity and elucidating their molecular regulatory mechanisms are of great practical significance for developing novel anti‐hepatic aging therapeutic strategies.

*Acorus tatarinowii*, a perennial herb belonging to the genus *Acorus* in the Araceae family, is a commonly used traditional Chinese medicine for the clinical treatment of cognitive dysfunction and neurological disorders, with a long history of rhizome‐based medicinal use. *Acorus tatarinowii* polysaccharides (ATP) are the primary active component of *Acorus tatarinowii*, and have been demonstrated to exert neuroprotective effects by alleviating oxidative stress, protecting mitochondrial function, and inhibiting the toll‐like receptor 4 (TLR4)/myeloid differentiation primary response 88 (MyD88)/nuclear factor kappa B (NF‐κB) pathway to improve age‐related neuroinflammation [21]. Meanwhile, ATP has also been reported to alleviate inflammatory bone loss [22]. The core pathological mechanisms of liver aging include chronic inflammation, oxidative stress, and mitochondrial dysfunction [3]. Although current studies on ATP have primarily focused on models of neuroinflammation and bone loss, its anti‐inflammatory, antioxidant, and mitochondrial protective effects are highly consistent with the core pathological processes of liver aging. Notably, *Acorus tatarinowii* has also demonstrated hepatoprotective effects against alcoholic liver disease and carbon tetrachloride (CCl_4_)‐induced hepatic injury [23, 24], suggesting that ATP may possess potential value in combating liver aging. However, the active components and mechanisms underlying the anti‐aging effects of ATP in the liver remain to be elucidated.

To address this issue, the present study aims to characterize the physicochemical properties of ATP, including their monosaccharide composition and molecular weight (MW), using chromatographic and spectroscopic techniques. Subsequently, network pharmacology and bioinformatics approaches will be employed to construct a “drug‐target‐disease” interaction network, thereby systematically elucidating the potential molecular mechanisms by which ATP may delay liver aging. Finally, molecular docking and a D‐galactose (D‐gal)‐induced liver aging rat model will be employed to further evaluate the therapeutic effects of ATP. This study aims to comprehensively elucidate the potential ameliorative effects of ATP on liver aging and to provide a theoretical basis for their development as natural anti‐aging drug candidates.

## 2 Materials and Methods

### 2.1 Plant Materials

Dried rhizomes of *Acorus tatarinowii* were purchased in July 2024 from Haozhou Kaiguan E‐Commerce Co., Ltd. (Anhui, China). Dr. Zhou Pengfei of the School of Basic Medicine at Guangdong Medical University conducted the species identification of the plant specimen. The plant specimen is housed in the Guangdong Medical University Gene Bank Herbarium, with specimen number GDD230XT81.

### 2.2 Extraction and Preparation of ATP

The rhizomes of *Acorus tatarinowii* were dried in an oven at 45°C, ground into powder using a grinder, and placed in a dialysis bag. Using hot water extraction and alcohol precipitation, the powder *of Acorus tatarinowii* was soaked in absolute ethanol (1:4, v/v) and ultrasonicated for 30 min to assist in pigment removal, and the procedure was repeated three times. Distilled water was then added at a ratio of 1:4 (w/v), and the mixture was stirred in a 100°C water bath for 2 h for extraction, repeated three times, and the filtrates were collected. The combined filtrate was concentrated under reduced pressure to 400 mL to obtain the concentrated polysaccharide extract [25]. Subsequently, proteins were removed using the Sevag method. Briefly, Sevag reagent (chloroform and *n*‐butanol mixed at a 4:1 ratio) was added, and the mixture was transferred to a separatory funnel, shaken vigorously for 10 min, and then centrifuged at high speed (4000 rpm, 20 min) to separate the layers. The middle protein layer and the lower organic phase were discarded. Collect the upper aqueous phase, and repeat the addition of Sevag reagent and removal of proteins three times in total. Under magnetic stirring, ethanol (95%, v/v) was slowly added dropwise to a final concentration of 80% (v/v), and the solution was left to stand overnight. After centrifugation at high speed (4000 rpm, 15 min) the next day, the supernatant and precipitate were separated. The precipitate was dried and lyophilized to obtain the crude polysaccharides of *Acorus tatarinowii*.

### 2.3 Structural Characterization of ATP

#### 2.3.1 Determination of ATP Monosaccharide Composition

Polysaccharides (5 mg) were hydrolyzed with 2 M trifluoroacetic acid at 121°C for 2 h in a sealed tube. The hydrolysate was evaporated to dryness under nitrogen, washed with methanol, and dried again [26]. The residue was redissolved in deionized water and filtered through a 0.22 µm membrane. Monosaccharide composition was determined by high‐performance anion‐exchange chromatography (HPAEC) using a Dionex CarboPac PA‐20 column, with data acquired via Chromeleon 7.2 CDS and quantified by the external standard method [27].

#### 2.3.2 Determination of ATP MW

The MW of ATP was determined using a GPS‐RI‐MALLS system [28]. ATP was dissolved in 0.1 mol/L NaNO_3_ to a final concentration of 1 mg/mL and reacted at 100°C for 20 min. After filtration through a 0.45 µm membrane, 100 µL of the sample was separated on tandem Shodex SB‐805 HQ and SB‐803 HQ gel columns at 45°C. The eluate was analyzed sequentially by a refractive index detector (RI) and a multi‐angle laser light scattering detector. Data were processed using ASTRA 6.1 software.

#### 2.3.3 Fourier Transform‐Infrared Spectroscopy (FT‐IR)

Dried polysaccharide (2 mg) was thoroughly mixed with potassium bromide powder at a ratio of 1:100 (w/w) and pressed into a 1 mm pellet. FT‐IR spectra were recorded in the range of 4000–400 cm^−1^ using a Fourier transform infrared spectrometer (Thermo Nicolet, USA) [29].

#### 2.3.4 SEM Analysis

The surface morphology of ATP was observed using a scanning electron microscope (SEM, Hitachi, Japan). The observations were conducted at an accelerating voltage of 2.0 kV and magnifications of 500×, 1000×, 2500×, and 5000×.

### 2.4 ATP Target Identification and Analysis

The SMILES identifiers of monosaccharides were obtained from the PubChem database. The SMILES were entered into the SwissTargetPrediction database (http://swisstargetprediction.ch/) to predict potential targets of each component. The predicted targets were pooled and deduplicated to obtain the final targets of ATP.

### 2.5 Acquisition of Liver Aging‐Related Gene Targets

The GSE133815 dataset (GPL570 platform) was selected from the Gene Expression Omnibus (GEO) database to obtain targets associated with hepatocyte senescence. Data were processed using the online platform (https://www.aclbi.com/), and differentially expressed genes (DEGs) were identified based on the criteria of *p* < 0.05 and |log2FC| ≥ 2.0. Volcano and heat maps were generated to visualize the results. Gene sets related to inflammation and oxidative stress were retrieved from the GeneCards database (https://www.genecards.org/).

### 2.6 Protein‐Protein Interaction (PPI) Network

The targets were imported into the STRING database (https://string‐db.org/) for PPI network analysis with a confidence score ≥ 0.90. The resulting network was visualized and analyzed in Cytoscape (v3.10.3), where nodes were ranked according to their degree values. Additionally, target clustering was performed using the Metascape database (https://metascape.org/gp/index.html#/main/step1) to identify Molecular Complex Detection (MCODE) cluster modules.

### 2.7 Gene Ontology (GO) and Pathway Enrichment Analysis

Enrichment analyses including GO, Kyoto Encyclopedia of Genes and Genomes (KEGG), and Reactome were performed using the DAVID database (https://david.ncifcrf.gov/), and KEGG‐enriched entries were categorized accordingly. Statistically significant enrichment results were visualized as bubble plots using the online platform (http://www.bioinformatics.com.cn/).

### 2.8 Gene Set Enrichment Analysis (GSEA)

GSEA was performed using the molecular signatures database (MSigDB) gene sets “c2.cp.kegg.v7.2.symbols.gmt” and “c2.cp.wikipathways.v7.4.symbols.gmt” as the reference. The “clusterProfiler” and “enrichplot” R packages (R v4.5.1) were used for functional annotation and visualization.

### 2.9 Weighted Gene Co‐Expression Network Analysis (WGCNA)

WGCNA was performed on the GSE133815 dataset using the “WGCNA” package (v1.71) in R software (version 4.5.1). An appropriate soft‐thresholding power was determined to construct a weighted adjacency network, followed by dynamic tree cut analysis to identify gene clusters. Modules significantly associated with liver aging were selected based on correlation analysis between module eigengenes and phenotypic traits. Module membership (MM) quantified the correlation between individual genes and the module eigengene, while gene significance (GS) reflected the correlation between each gene and the liver aging phenotype.

### 2.10 Machine Learning

To further screen the core targets, three machine learning algorithms were applied to the 18 hub genes. The ‘glmnet’, ‘e1071’, and ‘xgboost’ R packages were used to implement Least Absolute Shrinkage and Selection Operator (LASSO) regression, Random Forest (RF), and eXtreme Gradient Boosting (XGBoost), respectively. Finally, the intersection of the results from the three algorithms was taken to identify the final core genes.

### 2.11 Evaluation of the Diagnostic Potential of Key Genes

The expression levels of the key genes in the dataset were displayed using box plots. Receiver operating characteristic (ROC) analysis for the key genes was performed and visualized using the “glm” function and the “pROC” package in R, constructing a logistic regression model. The area under the curve (AUC) was used to evaluate the diagnostic validity of the potential liver aging biomarkers.

### 2.12 Immune Cell Infiltration and Single‐Cell Sequencing Analysis

The overall infiltration levels of 26 immune cell types in each sample were represented by the enrichment scores obtained via the Single‐sample Gene Set Enrichment Analysis (ssGSEA) algorithm in the “GSVA” R package. Spearman correlation analysis was performed to assess the relationships between the core genes and immune cells. Finally, single‐cell sequencing data and immunohistochemistry results from the Human Protein Atlas (HPA) database (https://www.proteinatlas.org/) were used to validate these findings.

### 2.13 Molecular Docking

The 3D structures of the target proteins were obtained from the Protein Data Bank (https://www.rcsb.org/). The 3D structure files of the individual monosaccharides of ATP were downloaded from PubChem. These structures were uploaded to the CB‐Dock2 online molecular docking platform (https://cadd.labshare.cn/cb‐dock2/php/index.php) to perform the docking calculations. Finally, the docking results between the components and the targets were visualized using PyMOL (v3.1.6.1).

### 2.14 Animal Experiment Validation

#### 2.14.1 Animals and Experimental Protocol

The experiment was conducted based on the D‐gal‐induced aging rat model protocol established in our previous study [30]. Fifteen 8‐week‐old male Sprague‐Dawley (SD) rats were selected and housed under standard conditions with a 12‐hour light/dark cycle at 22 °C ± 2 °C and 55 ± 5% humidity. All animal experiments were conducted in accordance with the *National Research Council's Guide for the Care and Use of Laboratory Animals* (National Institutes of Health Publications No. 8023, revised 2011) and the relevant Chinese regulations, including the *Regulations on the Management of Laboratory Animals* (Order No. 2, 1988). All procedures were approved by the Animal Ethics Committee of Guangdong Medical University (Approval No. SYXK 2024‐0213). After a 1‐week acclimation period, the animals were randomly divided into a control group, a model group, and an ATP group. The control group received subcutaneous injections of saline at the nape of the neck for 8 weeks. The model group was subcutaneously injected with D‐gal at 1000 mg/kg/day for 8 weeks. Rats in the ATP group were injected with the same dose of D‐gal for 8 weeks and, starting from the third week, were orally administered ATP at 200 mg/kg/day until the end of the experiment. This dosage was determined based on the commonly used dosage range of plant‐derived polysaccharides in D‐gal‐induced aging models [31, 32], and in conjunction with the preliminary exploratory findings from our research group, while control and model group rats received equivalent volumes of saline via the same route. Body weights were measured and recorded weekly throughout the experiment. At the end of the experiment, animals were fasted for 12 h, anesthetized with sodium pentobarbital, and euthanized, and serum and tissue samples were collected.

#### 2.14.2 Behavioral Tests of Morris Water Maze (MWM)

One week before the end of the experiment, the MWM spatial exploration test was conducted to evaluate the behavioral activity of the rats. The experimental setup consisted of a black circular pool (diameter 160 cm), a black circular platform, and an image acquisition system. The pool was divided into four equal quadrants, with the platform placed at the center of one quadrant. A camera was positioned above the pool to record data for each trial. Before each trial, water (25°C ± 2°C) was added to the pool, with the water level approximately 1.5 cm above the platform. After five days of the navigation training test, the platform was removed on the sixth day for the spatial exploration test. Rats were allowed to swim freely for 60 s, and a computer‐based video analysis system recorded their swimming distance, speed, and other related parameters.

#### 2.14.3 Preparation of Liver Tissue Sections

Rat liver tissues were fixed in 4% paraformaldehyde, followed by dehydration through a graded ethanol series, paraffin embedding, and sectioning into 5 µm thick slices. The tissue sections were deparaffinized and stained with hematoxylin and eosin (H&E). Immunohistochemical staining was performed to detect the protein expression of TLR4, NF‐κB, Nuclear factor‐erythroid 2‐related factor 2 (Nrf2), and Heme oxygenase‐1 (HO‐1) in the liver tissues. The specific experimental steps followed our previous study [30]. All images were captured randomly from five fields of view using an optical microscope. Positive signals in the immunohistochemical images were quantitatively analyzed using ImageJ software (v1.54p).

### 2.15 Statistical Analysis

All experimental data were analyzed using GraphPad Prism 9.0, and experiments were performed in triplicate to ensure result reliability. Data are presented as mean ± standard deviation. Differences between two groups were assessed using the t‐test or Mann–Whitney U test, while comparisons among multiple groups were conducted using one‐way analysis of variance (ANOVA). Statistical significance was set at *p* < 0.05.

## 3 Results

### 3.1 Structural Characterization of ATP

As shown in Figure 1A, the MW distribution curve of ATP showed a single and symmetrical peak shape, suggesting that its MW distribution was relatively homogeneous. The results showed that the weight‐average molecular weight (Mw) and number‐average molecular weight (Mn) of ATP were 159.099 kDa and 56.093 kDa, respectively. The analysis of the composition of monosaccharides showed that ATP was mainly composed of Rhamnose (Rha), Arabinose (Ara), Galactose (Gal), Glucose (Glc), Xylose (Xyl), and Galacturonic acid (GalA), with molar ratios of 2.98: 9.11: 4.93: 72.63: 5.85: 4.49. Among them, Glc (74.4%), Ara (7.8%), and Xyl (5.0%) were the major monosaccharide components (Figure 1B). SEM observations at different magnifications showed that the ATP exhibits an asymmetric flaky porous structure with rough aggregates scattered on its surface, and irregular textures in local regions (Figure 1C). The ATP was further structurally characterized using FT‐IR. A strong hydroxyl (─OH) stretching vibration peak was observed at 3379.14 cm^−1^, and a weaker C─H stretching vibration peak appeared at 2932.8 cm^−1^. The absorption peak at 1735.27 cm^−1^ can be attributed to the C═O stretching vibration of uronic acid [33]. Characteristic peaks at 1621 and 1424 cm^−1^ corresponded to the asymmetric and symmetric stretching vibrations of COO^−^ groups [34]. In addition, absorption peaks at 1375 and 1245.5 cm^−1^ were related to C─H bending vibrations. Peaks at 1152.7, 1079.7, and 1020 cm^−1^ were assigned to C─O─C and C─O─H stretching vibrations of the pyranose ring, while the peak at 861 cm^−1^ indicated the presence of α‐glycosidic linkages in ATP (Figure 1D).

**FIGURE 1:**
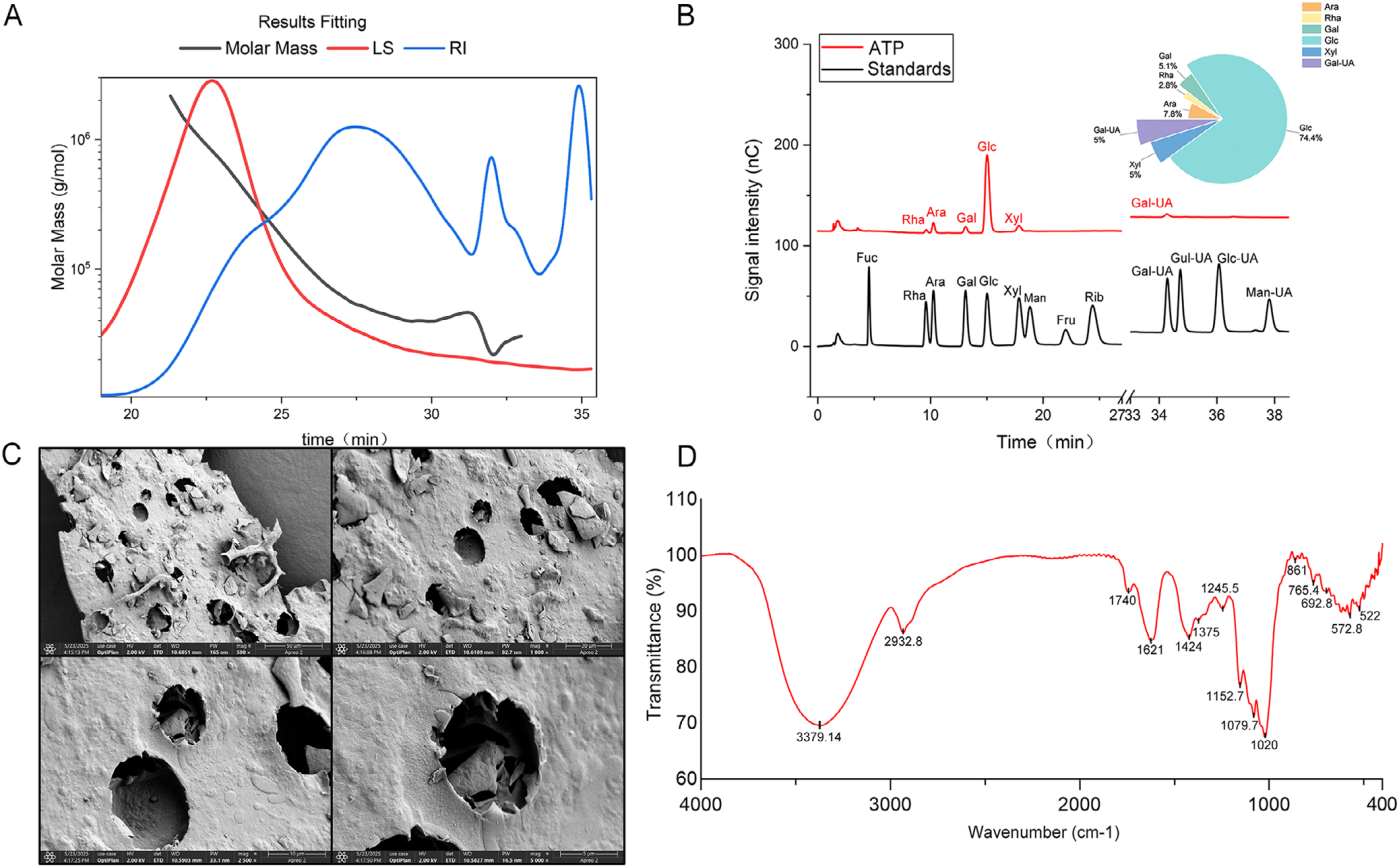
Structural Characterization of ATP. (A) MW determination of ATP. (B) Chromatograms of standard monosaccharides and ATP monosaccharides as well as pie charts of the molar percentage composition of ATP monosaccharides. (C) SEM images at different magnifications. (D) FI‐IR analysis of ATP.

### 3.2 Screening and Functional Enrichment Analysis of DEGs

Differential expression analysis of the GSE133815 dataset identified 3,147 DEGs, which were visualized using volcano and heat maps (Figure 2A,B). These results indicated distinct gene expression differences between the two groups. KEGG enrichment analysis of the DEGs was performed to explore the potential mechanisms underlying liver aging. The results showed that upregulated genes were mainly enriched in pathways such as the NF‐κB signaling pathway (Figure 2C), whereas downregulated genes were primarily enriched in the mTOR signaling pathway and autophagy (Figure 2D). GSEA was conducted to examine enrichment in WikiPathways and KEGG. KEGG enrichment results showed significant upregulation of “Fatty acid metabolism,” “Glycolysis/Gluconeogenesis,” and “MAPK signaling pathway” in the aging group compared to the young group (Figure 2E). According to WikiPathways enrichment, “Oxidative stress response” and “Inflammatory response pathway” were significantly upregulated in the aging group relative to the young group (Figure 2F).

**FIGURE 2:**
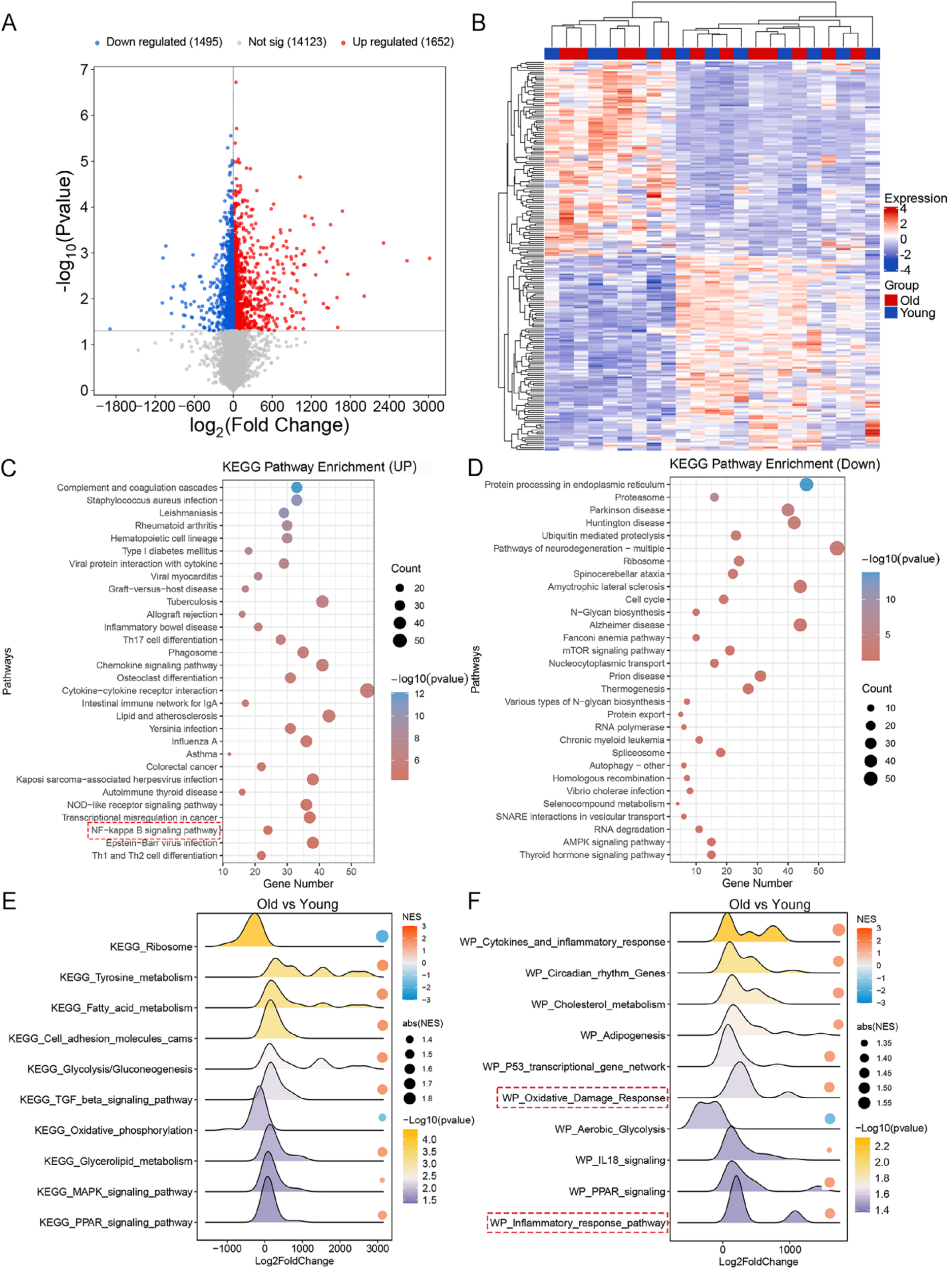
Screening of DEGs and their enrichment analysis. (A) Volcano plot of DEGs. (B) Heatmap of DEGs. (C) Bubble plot of KEGG enrichment for upregulated genes. (D) Bubble plot of KEGG enrichment for downregulated genes. P‐values indicate the significance of enrichment. (E) GSEA enrichment of KEGG pathways. (F) GSEA enrichment of WikiPathways. Bubble color corresponds to the NES value, and the peaks represent the expression levels of the corresponding gene sets.

### 3.3 Network Pharmacology Combined With Bioinformatics Analysis of the Anti‐Liver Aging Mechanism of ATP

#### 3.3.1 PPI Network of ATP as a Potential Target for Anti‐Liver Aging

A total of 1,333 potential targets of ATP were predicted using the SwissTargetPrediction database. An intersection analysis of ATP and DEGs using Venn revealed 272 common targets (Figure 3A). PPI analysis of these 272 targets was performed to investigate their interactions. A PPI network was constructed using the STRING database, and the degree of each target was calculated in Cytoscape and ranked accordingly. The top‐ranked targets included TP53, IL6, TLR4, NFKB1, IL1B, and AKT1 (Figure 3B). Clustering analysis of the 272 targets using the Metascape database identified eight MCODE modules (Figure 3C).

**FIGURE 3:**
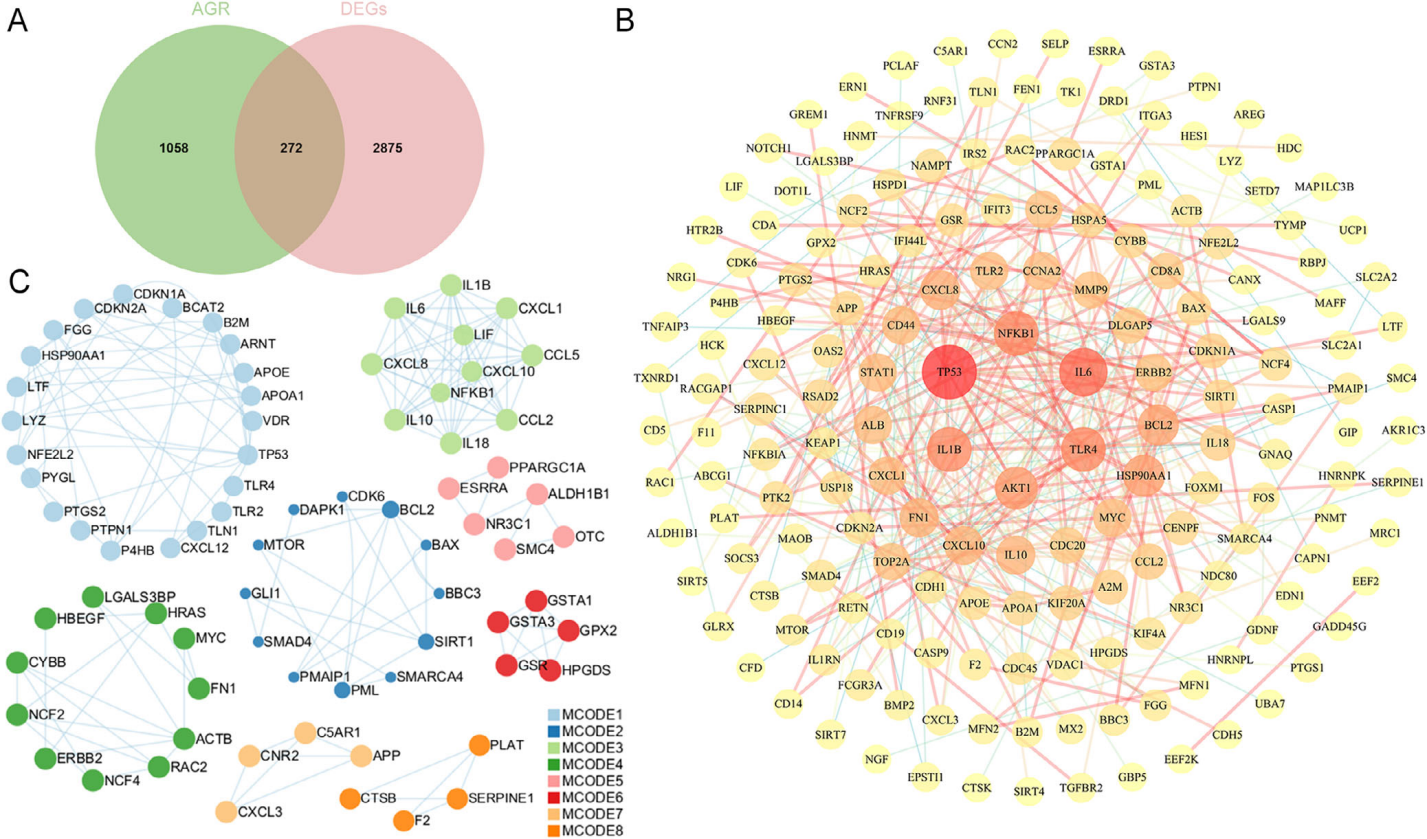
PPI network of ATP as a potential target for anti‐liver aging. (A) Intersection analysis of ATP targets and targets of liver aging DEGs. (B) PPI network of 272 potential targets of ATP anti‐liver aging. (C) A total of 8 MCODE clusters were obtained from clustering analysis of 272 targets.

#### 3.3.2 Enrichment Analysis of Intersection Targets and Elucidation of Anti‐Inflammatory and Antioxidant Mechanisms

Enrichment analysis of 272 potential targets was performed using the DAVID database. KEGG pathway analysis identified 129 significantly enriched pathways. Among them, targets in the Organismal Systems category were primarily enriched in the Toll‐like receptor signaling pathway, while those in the Cellular Processes category were notably enriched in Apoptosis and Cellular senescence. In Reactome Pathway analysis, targets were mainly concentrated in the KEAP1‐NFE2L2 pathway and Toll‐like Receptor Cascades (Figure 4A). GO enrichment analysis showed that in the Biological Process (BP) category, targets were mainly enriched in inflammatory response and response to oxidative stress; in the Cellular Component (CC) category, enrichment was observed in extracellular space and extracellular region; and in the Molecular Function (MF) category, targets were primarily enriched in protein binding and signaling receptor binding (Figure 4B). A “pathway—target—component” Sankey diagram was constructed for visualization (Figure 4C).

**FIGURE 4:**
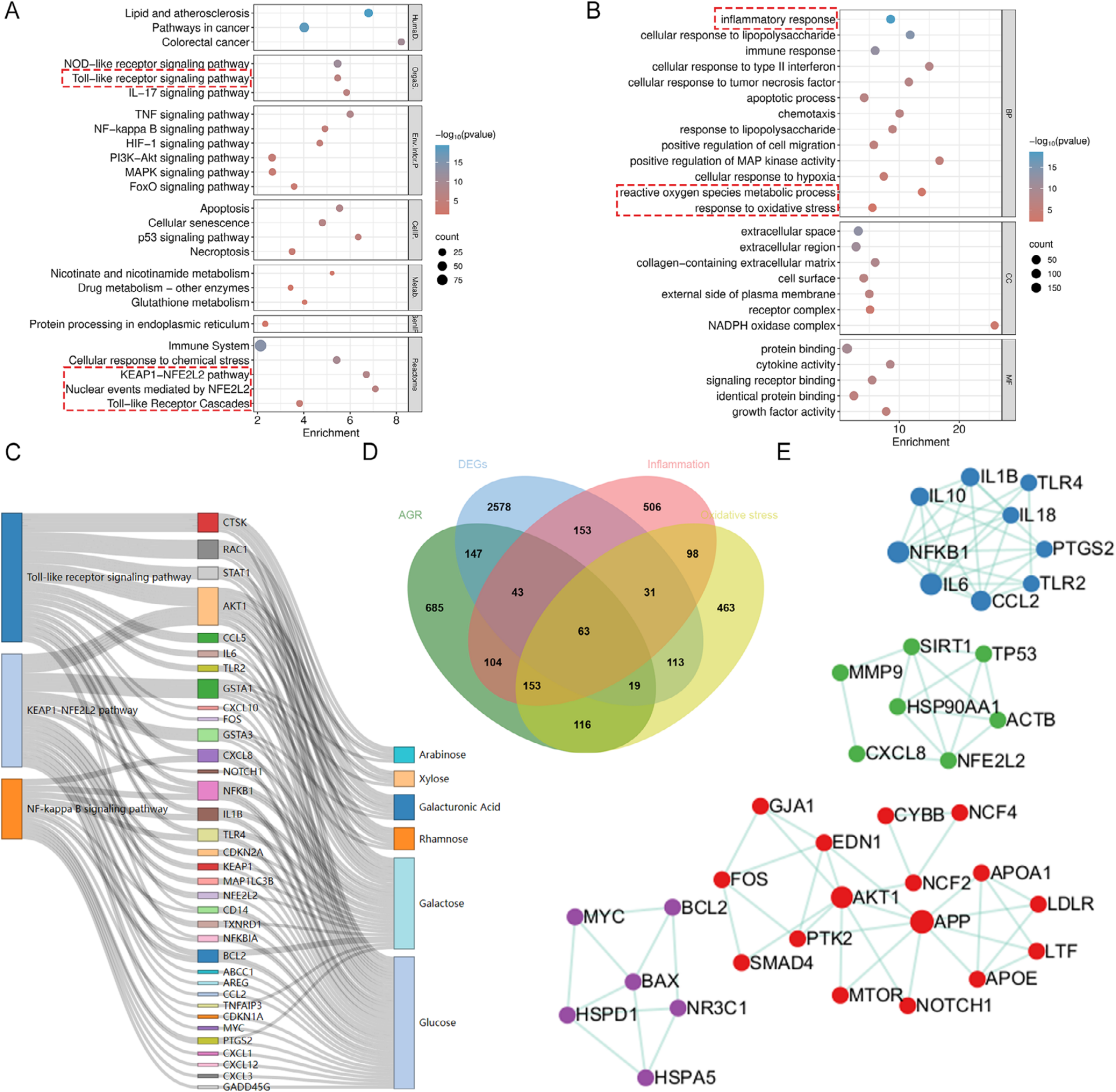
Enrichment analysis of intersecting targets. (A) Bubble plot of pathway enrichment analysis. (B) Bubble plot of GO enrichment analysis. (C) Sankey diagram. (D) Venn diagram of ATP targets, DEGs, inflammation‐related, and oxidative stress‐related targets. (E) The 63 common targets clustered into 4 MCODE modules.

A total of 1,151 inflammation‐related genes and 1,056 oxidative stress‐related genes were obtained from the GeneCards database. Venn analysis of ATP targets with these gene sets identified 63 common targets (Figure 4D). Metascape analysis of these 63 hub genes revealed four MCODE clusters (Figure 4E). Collectively, these results indicate that the pharmacological effects of ATP against liver aging are primarily mediated through inflammation‐ and oxidative stress‐related mechanisms and pathways.

#### 3.3.3 WGCNA

To comprehensively analyze the key genes associated with liver aging, this study applied WGCNA to screen gene modules most closely correlated with the liver aging samples. Based on scale independence and average linkage, a soft threshold of 16 was selected (Figure 5A). WGCNA generated 21 distinct gene modules, visualized using a dendrogram (Figure 5B). We further analyzed the correlation between gene modules and the aging phenotype, revealing that the red module (*r* = 0.56, *p* = 0.005) and blue module (*r* = 0.7, *p* = 2e‐04) were significantly positively correlated with liver aging (Figure 5C–E). Accordingly, genes from the red and blue modules were combined to represent the WGCNA results. Finally, to further screen for key genes targeted by ATP in liver aging, the 63 hub genes were intersected with the key module genes, yielding 18 genes for subsequent analyses (Figure 5F).

**FIGURE 5:**
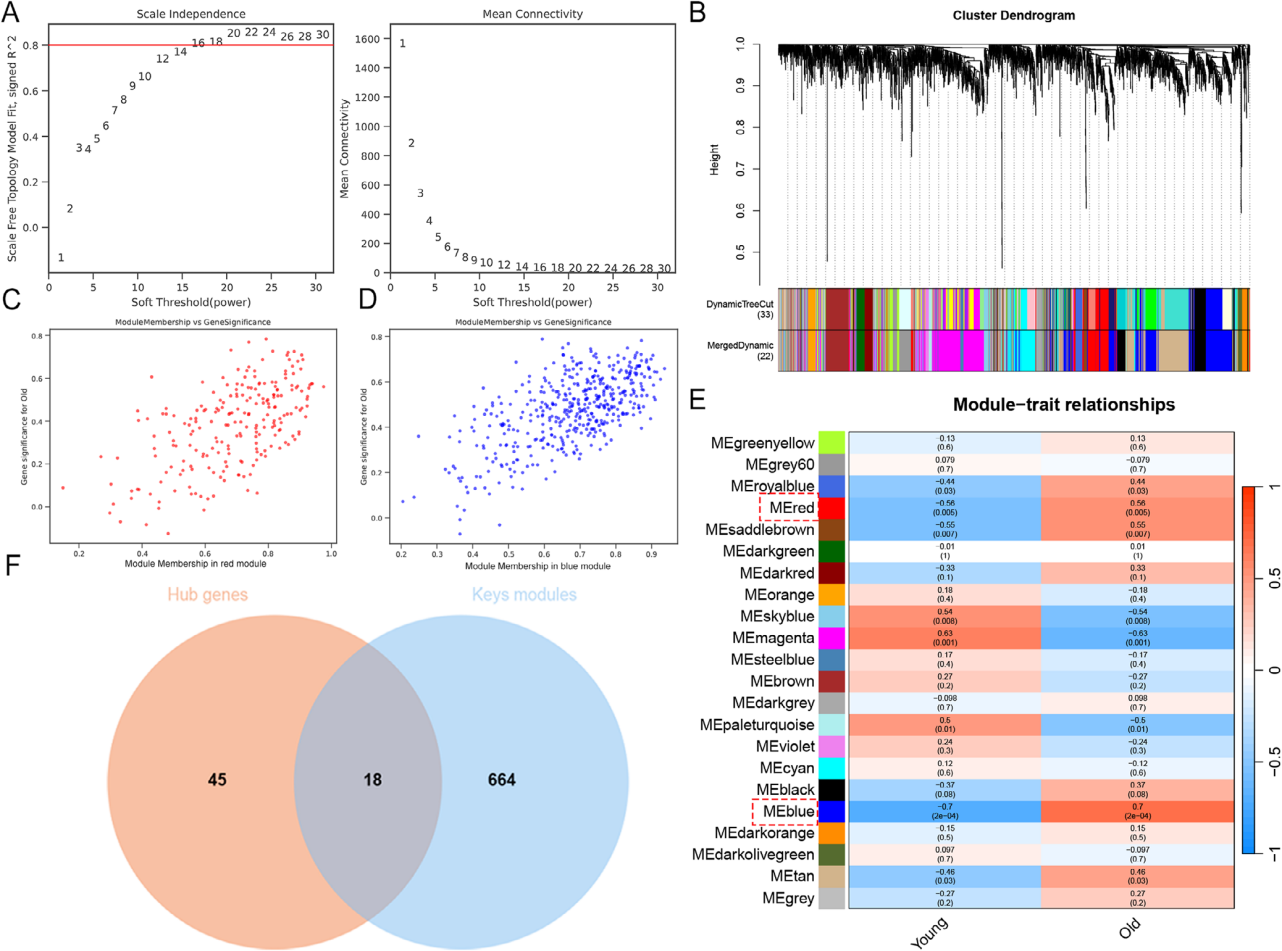
Identification of Key Genes for ATP Intervention in Liver Aging by WGCNA. (A) Scale‐free topology analysis of WGCNA (*β* = 16). (B) Gene dendrogram and modules generated by WGCNA. (C–D) GS–MM relationship plots for the red and blue modules associated with liver aging. (E) Heatmap showing correlations between gene modules and phenotype. (F) Venn diagram of the intersection between hub genes and key module genes.

#### 3.3.4 Machine Learning Algorithms for Screening Core Targets

Three machine learning algorithms were constructed based on 18 hub genes to screen the core targets of ATP for the treatment of liver aging. The LASSO regression screened five targets including CASP1, FAS, IL1RN, NFE2L2, and TLR4 (Figure 6A). The RF model screening yielded nine targets, of which TLR4, CASP1, NFE2L2, and NAMPT had relative importance scores greater than 1 (Figure 6B). The XGBoost algorithm screened seven feature genes, and the AUC = 0.9667 indicated a good model fit (Figure 6C). The genes common to the three algorithms, CASP1, NFE2L2, and TLR4, were identified using the Venn diagram (Figure 6D). ROC curve analysis was performed to validate the reliability of the results. The results showed that the AUC values of these genes were greater than 0.8. CASP1, NFE2L2, and TLR4 had high accuracy and specificity in distinguishing between the two groups of samples (Figure 6E). In addition, to verify the accuracy of the final core genes, we examined their expression levels in the GSE133815 dataset. The results showed that TLR4, NFE2L2, and CASP1 were significantly up‐regulated in the senescent group compared to the young group, suggesting their potential roles in liver aging (Figure 6F). Notably, the machine learning algorithms in this study serve only as complementary target‐screening tools rather than predictive models.

**FIGURE 6:**
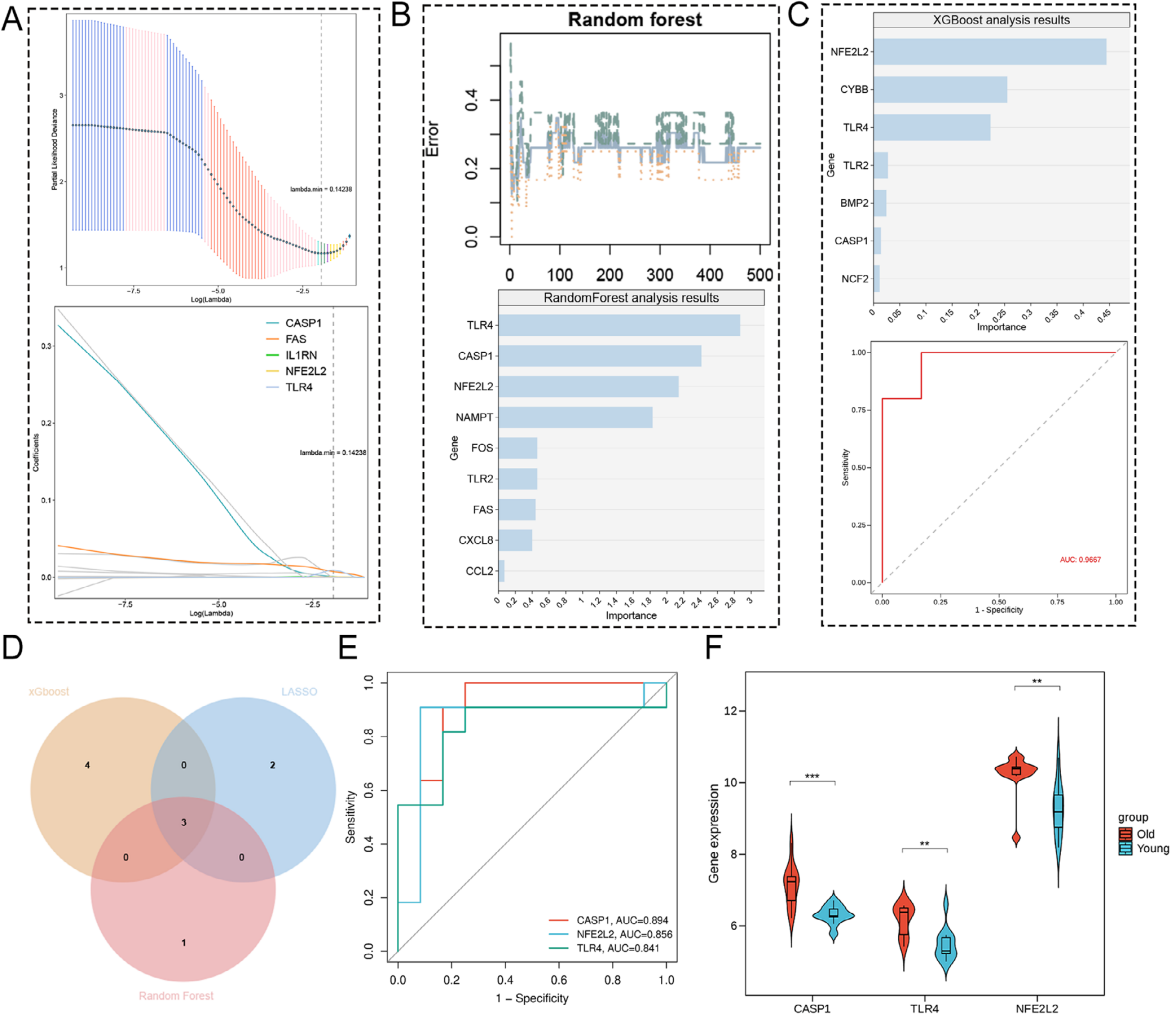
Machine learning screening of core targets. (A) LASSO regression cross‐validation curves and LASSO coefficient pathway plots for key genes. (B) RF tree and RF plot to calculate gene importance maps. (C) XGBoost analysis results and XGBoost model ROC plot. (D) Venn diagram of the intersection of the regression methods. (E) ROC curve of CASP1, TLR4, and NFE2L2 and TLR4 genes. (F) Statistical plots of CASP1, TLR4, and NFE2L2 and TLR4 gene expression levels. Data are presented as mean ± SD. ^*^ *p* < 0.05, ^**^ *p* < 0.01, ^***^ *p* < 0.001.

#### 3.3.5 Immune Cell Infiltration and Single‐Cell Sequencing Analysis

The ssGSEA algorithm was used to evaluate immune cell infiltration in liver aging samples. Boxplots depict the comparison of immune infiltration scores between the two groups (Figure 7A). Compared with the young group, the aging group exhibited significantly increased levels of macrophages, myeloid‐derived suppressor cells (MDSCs), and mast cells (*p* < 0.05). Spearman correlation analysis indicated that the key genes were positively correlated with immune cells, suggesting their potential role in modulating the immune microenvironment. Notably, macrophage levels showed significant positive correlations with HMOX1, NFE2L2, NFKB1, TLR4, and CASP1 (*p* < 0.05) (Figure 7B). HMOX1 was found to be closely associated with macrophages in aging liver tissue. Further validation using single‐cell RNA‐seq data from the HPA database showed that HMOX1 is predominantly expressed in Kupffer cells in human liver tissue (Figure 7C). In addition, immunohistochemistry data from the HPA database revealed age‐related differences in HO‐1 protein expression: in liver tissue from a 55‐year‐old individual, HO‐1 was mainly localized in Kupffer cells within the hepatic sinusoids, whereas in a 79‐year‐old individual, HO‐1 was expressed in both hepatocytes and Kupffer cells. HO‐1 expression in hepatocytes increased markedly with age, suggesting that HO‐1 expression in the liver may be age‐dependent (Figure 7D).

**FIGURE 7:**
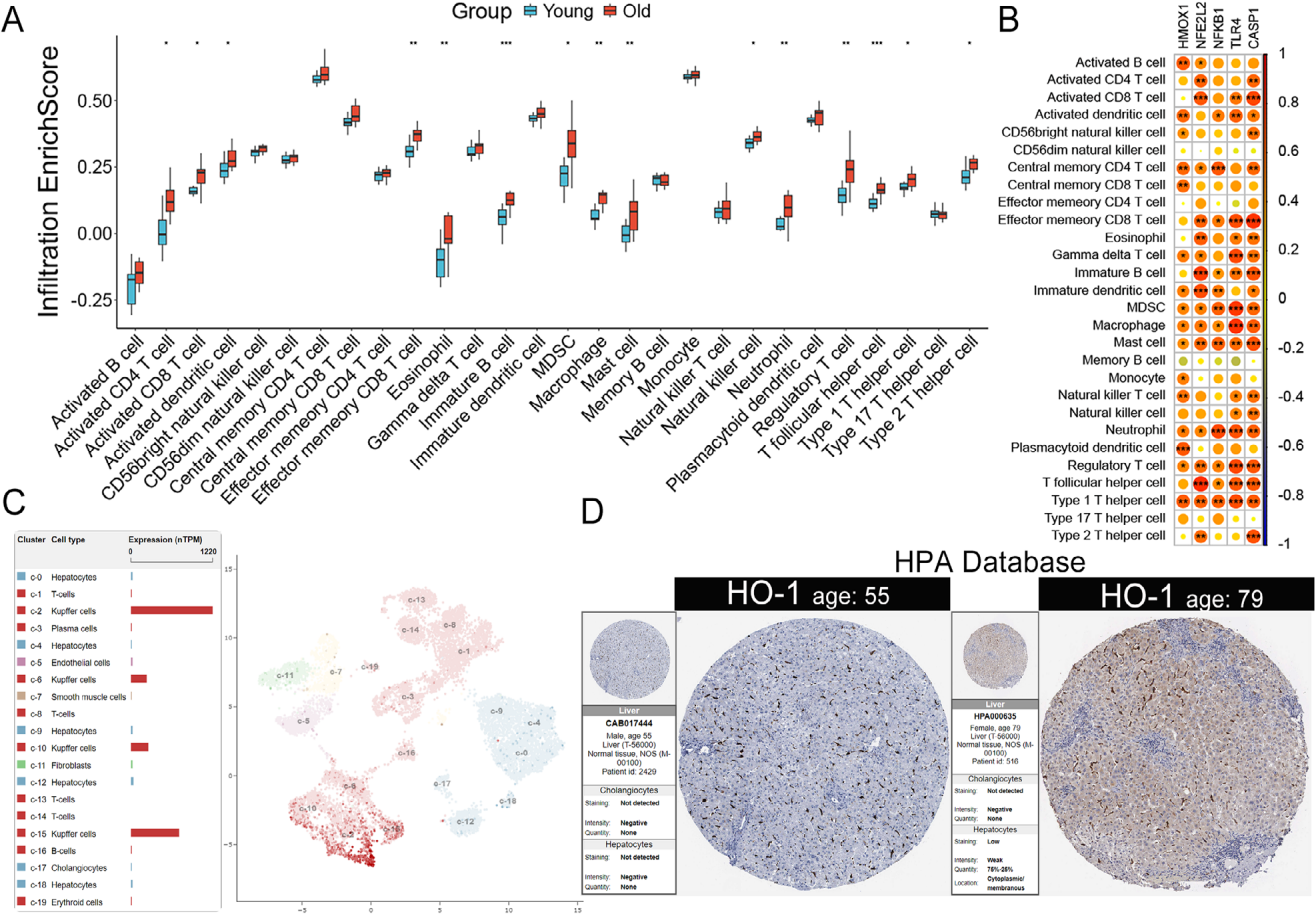
Immune infiltration analysis and single‐cell sequencing. (A) Box plots showing the immune infiltration scores of the two groups. (B) Correlation analysis between core genes and immune cell infiltration, with red and blue representing positive and negative correlation, respectively. (C) Expression and analysis of the gene HMOX1 in single‐cell RNA sequencing data. (D) Immunohistochemical staining of HO‐1 in the normal liver of different ages. Data are presented as mean ± SD. ^*^ *p* < 0.05, ^**^ *p* < 0.01, ^***^ *p* < 0.001.

### 3.4 Molecular Docking

In this study, the CB‐Dock2 molecular docking platform was used to estimate the binding energies between six monosaccharides and major target proteins, where lower binding energy indicates greater structural stability. As shown in Figure 8A, the heatmap illustrates the docking scores between each monosaccharide and the target proteins. The complexes with the lowest binding energies were Glc–Nrf2, Rha–Nrf2, Gal–Nrf2, and GalA–TLR4. The docking results further indicated that ATP monosaccharides exhibited strong binding affinities with Nrf2, TLR4, P65, and HO‐1 (binding energy less than –5.0 kcal/mol). Therefore, Nrf2, TLR4, P65, and HO‐1 may serve as key targets through which ATP exerts its anti‐liver aging effects. Notably, molecular docking data merely represent predictive hypotheses regarding the potential target‐pathway associations of ATP.

**FIGURE 8:**
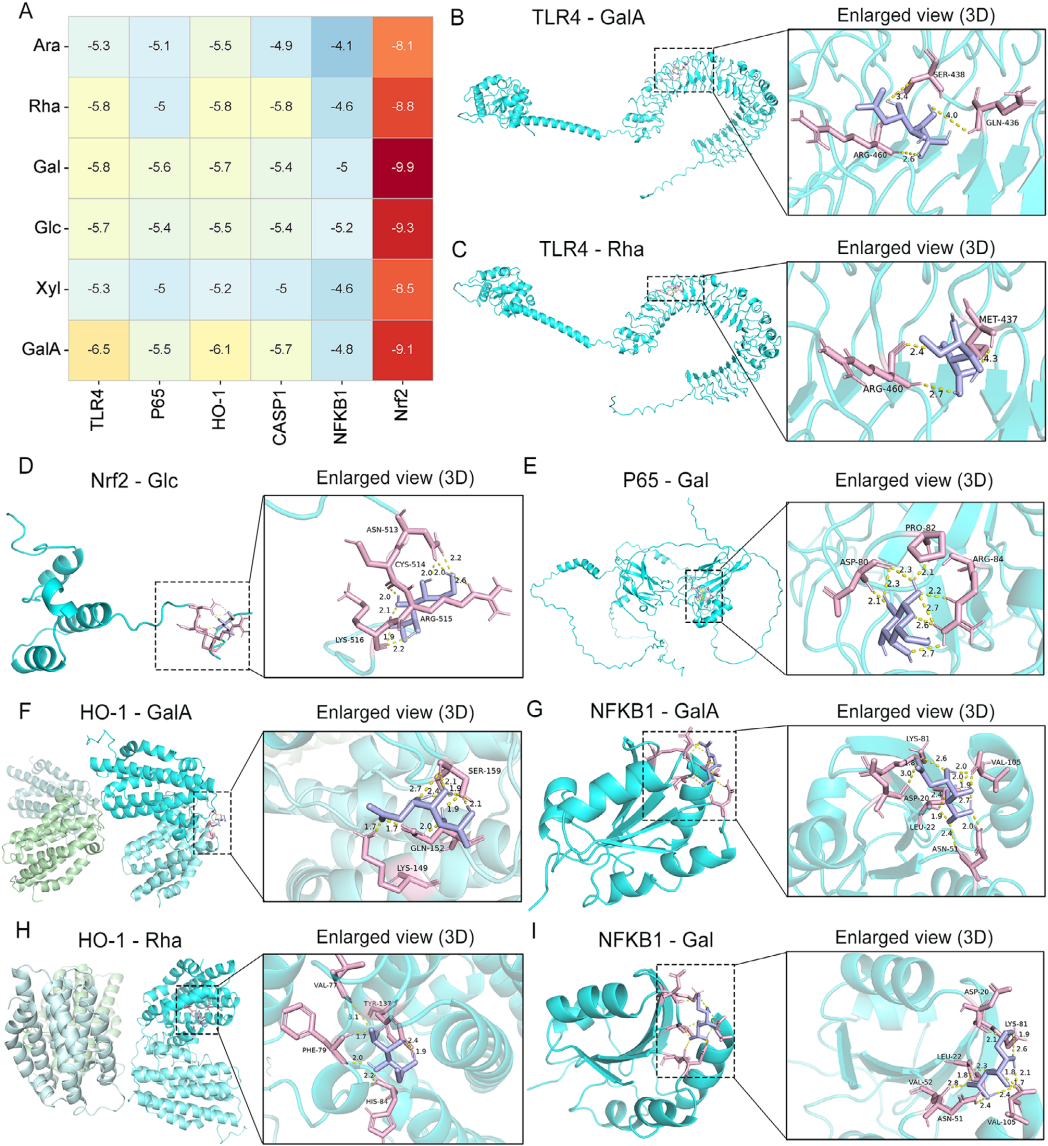
Molecular docking results. (A) Heatmap of molecular docking scores. (B–I) Visualization of interaction modes between key target proteins and monosaccharides.

### 3.5 Animal Experimentation Validation

#### 3.5.1 General Conditions of the Rats

During the experiment, the appearance and activity of the rats were monitored. As shown in Figure 9B, compared with the control group, rats in the model group exhibited lethargy, reduced activity, sparse hair loss, partial yellowing of the fur, and a hunched posture with spinal curvature, indicating successful establishment of the aging model. After ATP treatment, compared with the model group, the rats exhibited improved mental state, increased activity, and glossier fur, effectively alleviating hair loss and dryness, indicating that ATP exerts a positive anti‐aging effect. Body weights increased gradually in all groups, with the model group showing lower overall weights than the control group, while the ATP group showed no significant difference from the control (Figure 9C). Food intake progressively increased in all groups, with the model group consuming less than the control and ATP groups, while ATP treatment maintained intake comparable to control levels (Figure 9D). Locomotor activity parameters (total swimming distance, maximum swimming speed, average swimming speed) showed no significant differences among groups. However, overall, the model group displayed slightly reduced locomotor activity compared with the control group, while the ATP group exhibited higher activity than the model group (Figure 9E–G).

**FIGURE 9:**
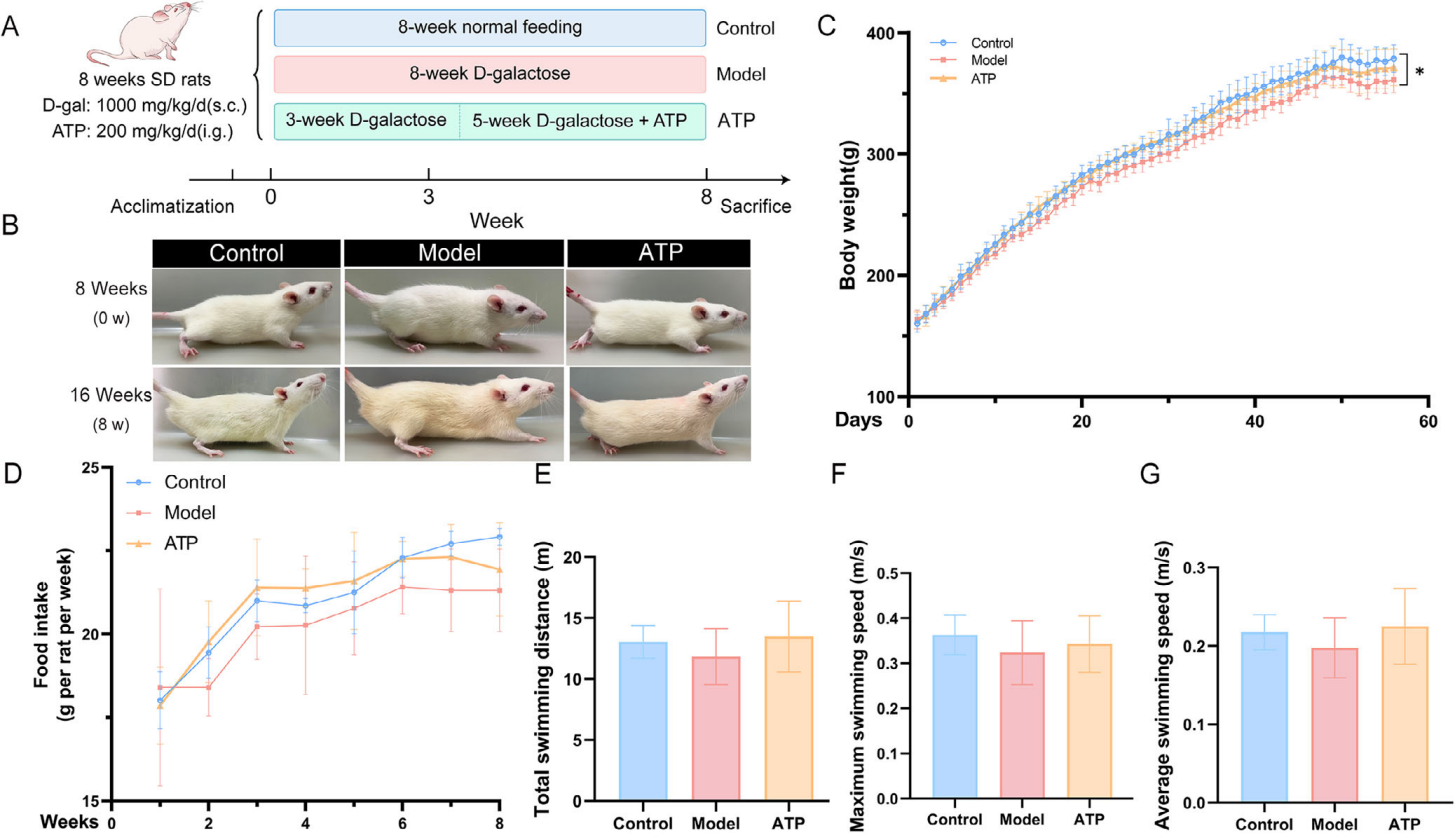
Effects of ATP on appearance, body weight, food intake, and activity of rats. (A) Schematic diagram of the animal experimental design. (B) Appearance of rats in each group. (C) Changes in body weight of rats in each group. (D) Changes in food intake of rats in each group. (E) Total swimming distance within 60 s. (F) Maximum swimming speed within 60 s. (G) Average swimming speed within 60 s. Data are presented as mean ± SD. ^*^ *p* < 0.05、^**^ *p* < 0.01 and ^***^ *p* < 0.001.

#### 3.5.2 Effects of ATP on Hepatic Histopathological Changes

The effects of ATP on hepatic pathological changes were evaluated by H&E staining, with results shown in Figure 10. In the control group, the liver tissue exhibited a clear architecture, with hepatocytes of uniform size and regular morphology. Hepatic cords were radially arranged around the central vein, and few hepatocytes showed signs of degeneration or necrosis. In contrast, the model group displayed typical features of hepatic aging, including disorganized hepatic cords, widened sinusoids, shrunken and irregular hepatocytes with indistinct boundaries, lipid degeneration and droplet accumulation, inflammatory cell infiltration, nuclear pyknosis, and increased necrosis. In the ATP‐treated group, these aging‐related histological abnormalities were alleviated. ATP therapy reduced sinusoidal and cord lesions in the liver, improved hepatocyte size and morphology, alleviated hepatic steatosis and necrosis, and decreased inflammatory cell infiltration. These results indicate that ATP treatment ameliorated hepatic aging and restored the liver condition to near‐control levels.

**FIGURE 10:**
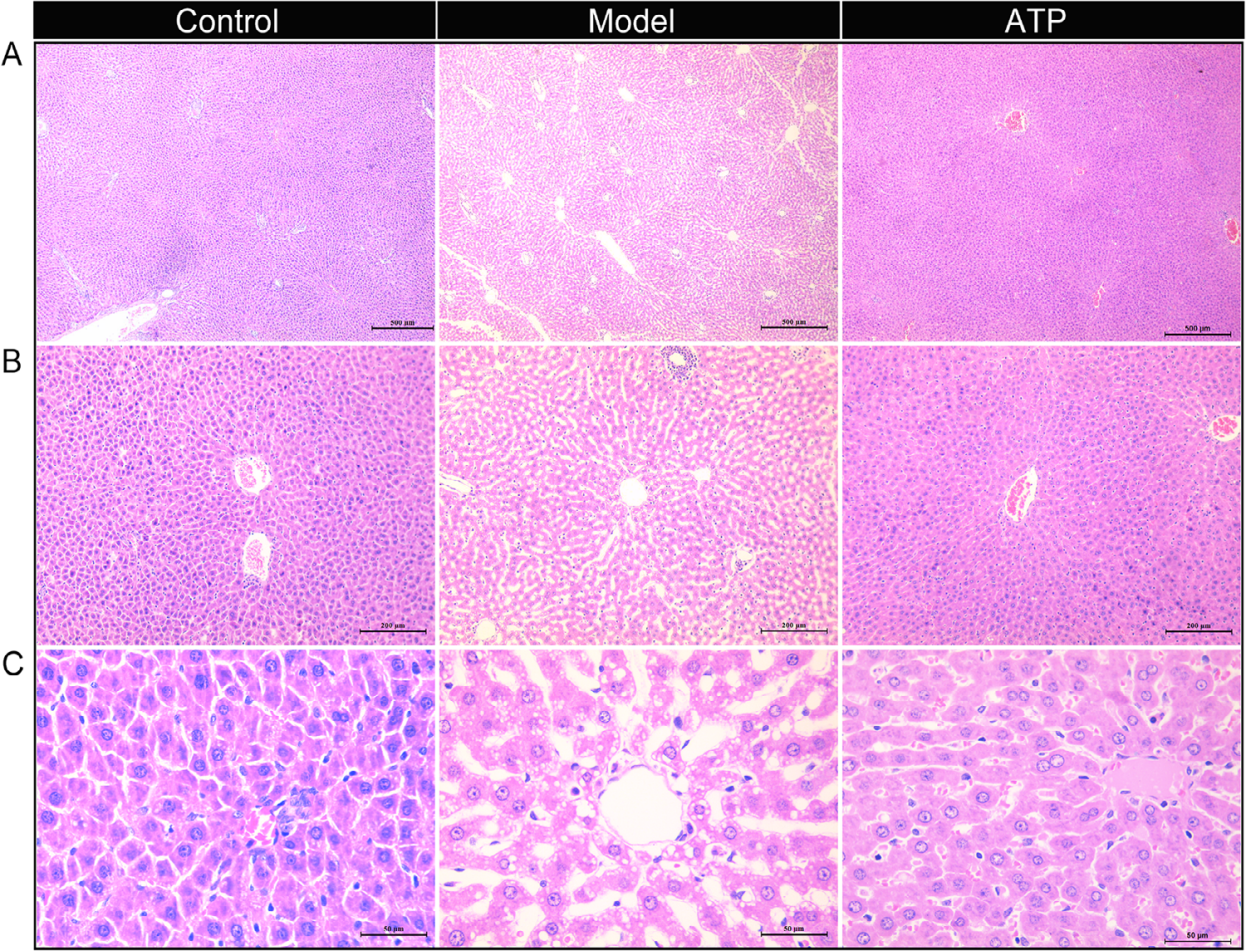
Effects of ATP on hepatic histopathology. (A) H&E staining of liver tissue, bar = 500 µm (×40). (B) H&E staining of liver tissue, bar = 200 µm (×100). (C) H&E staining of liver tissue, bar = 50 µm (×400).

#### 3.5.3 Regulation of TLR4, p‐NF‐κB, Nrf2, and HO‐1 Proteins in Liver Tissue by ATP

Based on the results of network pharmacology and machine learning analyses, TLR4, NFKB1, and NFE2L2 were identified as potential key targets of ATP in alleviating hepatic aging. To validate these predictions, immunohistochemical staining was performed to examine the expression of these proteins in liver tissues. As shown in Figure 11, the expression levels of TLR4 and p‐NF‐κB were markedly increased in the model group (*p* < 0.001). Following ATP treatment, the expression of TLR4 and p‐NF‐κB proteins was significantly downregulated (*p* < 0.001, *p* < 0.01), suggesting that ATP may attenuate hepatic aging by suppressing TLR4 activation and its downstream signaling molecule p‐NF‐κB, thereby exerting anti‐inflammatory effects (Figure 11).

**FIGURE 11:**
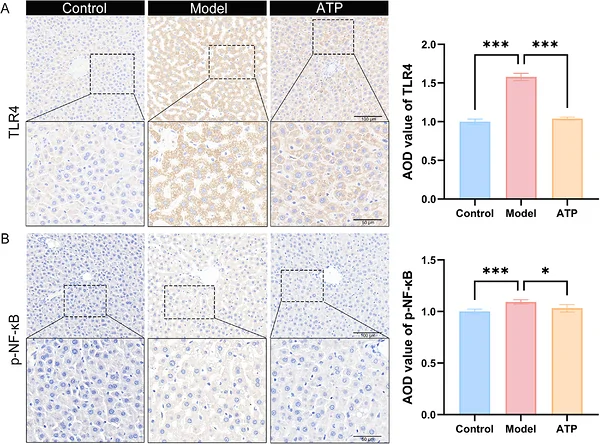
Effects of ATP on the expression of TLR4 and p‐NF‐κB proteins in the liver tissues of aging rats. (A) Representative immunohistochemical images and quantitative analysis of TLR4 expression in liver tissues. (B) Representative immunohistochemical images and quantitative analysis of p‐NF‐κB expression in liver tissues. Magnifications: ×200 (scale bar = 100 µm) and ×400 (scale bar = 50 µm). Data are presented as mean ± SD. ^*^ *p* < 0.05、^**^ *p* < 0.01 and ^***^ *p* < 0.001.

As shown in Figure 12A, the expression of Nrf2 protein was significantly decreased in the model group (*p* < 0.001). After ATP treatment, Nrf2 expression increased, indicating that ATP may exert antioxidant effects by activating the Nrf2 signaling pathway to enhance antioxidant defense capacity. To further explore the role of the Nrf2 pathway, we examined the expression of its downstream protein HO‐1. As shown in Figure 12B, HO‐1 expression was markedly elevated in the model group (*p* < 0.001), predominantly localized in Kupffer cells, whereas only weak, patchy staining was observed in a small number of hepatocytes. Following ATP intervention, HO‐1 expression levels were restored to near‐normal conditions, indicating that ATP may alleviate excessive stress responses and improve hepatic aging phenotypes by modulating the Nrf2/HO‐1 signaling balance. These findings further support the results of transcriptomic and network pharmacology analyses (Figure 12).

**FIGURE 12:**
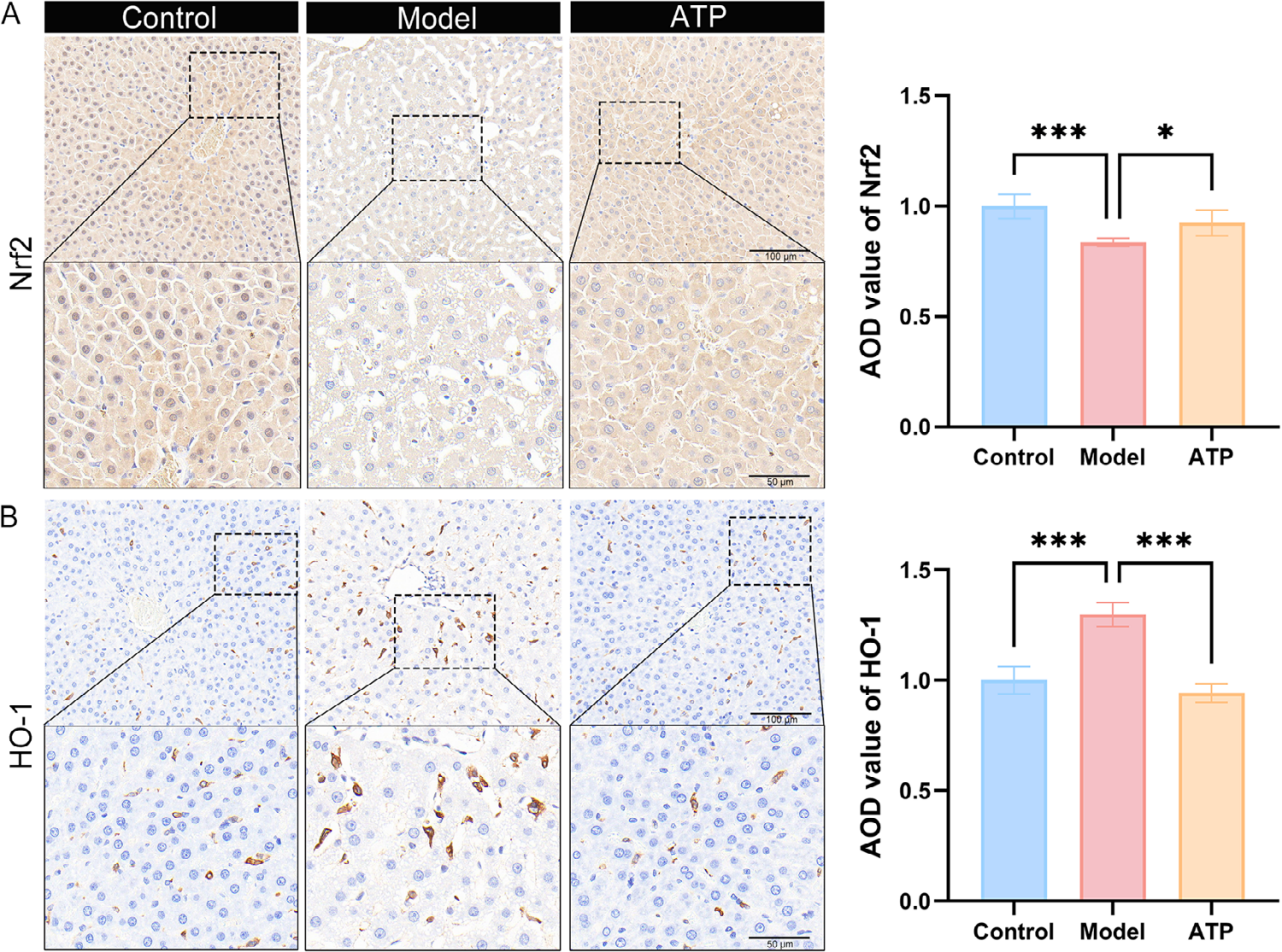
Effects of ATP on the expression of Nrf2 and HO‐1 proteins in the liver tissues of aging rats. (A) Representative immunohistochemical images and quantitative analysis of Nrf2 expression in liver tissues. (B) Representative immunohistochemical images and quantitative analysis of HO‐1 expression in liver tissues. Magnifications: ×200 (scale bar = 100 µm) and ×400 (scale bar = 50 µm). Data are presented as mean ± SD. ^*^ *p* < 0.05、^**^ *p* < 0.01 and ^***^ *p* < 0.001.

## 4 Discussion

Liver aging is a critical driver of systemic aging, with its core pathological mechanisms involving chronic inflammation, oxidative stress, and mitochondrial dysfunction. However, existing therapeutic approaches for liver aging have limitations in both efficacy and safety, highlighting the urgent need to explore novel, safe, and effective intervention strategies. In this context, polysaccharides derived from traditional Chinese medicines are considered promising hepatoprotective and anti‐aging candidates due to their antioxidative, anti‐inflammatory, mitochondrial function‐protective, autophagy‐regulating, and cellular senescence‐delaying effects [35, 36]. *Acorus tatarinowii*, a traditional Chinese medicine, has been demonstrated to possess potential hepatoprotective value, and its polysaccharide components exhibit anti‐inflammatory, antioxidative, and mitochondrial function‐protective activities. Nevertheless, the precise mechanisms by which ATP prevents liver aging remain not fully elucidated. In this study, by integrating structural characterization, network pharmacology, bioinformatics, and machine learning strategies, we for the first time reveal the protective effects of ATP against D‐gal‐induced liver aging from the perspectives of inflammatory response and oxidative stress, and predict TLR4 and Nrf2 as its core targets of action.

The bioactivity of polysaccharides is closely related to their structural characteristics, among which monosaccharide composition and MW are the two most important factors influencing polysaccharide bioactivity [37]. In this study, the predominant monosaccharides of ATP were Glc, Ara, Gal, GalA, and Rha, with an Mw of 159.1 kDa. Studies have demonstrated that the aforementioned monosaccharide components and Mw range are significantly associated with the antioxidative and anti‐aging activities of natural polysaccharides. In most cases, the monosaccharide constituents of polysaccharides with antioxidant effects mainly include Glc, Ara, Gal, GalA, and Man [38]. Li et al. reported that a higher proportion of Rha and Glc plays an important role in maintaining antioxidative and anti‐aging activities [39]. Furthermore, polysaccharides with higher uronic acid content exhibit favorable antioxidant activity [40]. Acidic polysaccharide CP‐2 from Dioscoreae Rhizoma, which possesses a relatively high proportion of GalA, can effectively ameliorate acute liver injury induced by alcohol [41]. Mw also affects the bioactivity of polysaccharides; however, its influence is not simply positively or negatively correlated [37]. It has been found that the Mw of polysaccharides with significant anti‐aging activity is predominantly concentrated within the range of 2–350 kDa [38]. In most instances, polysaccharides with lower Mw exhibit stronger anti‐aging activity than those with higher Mw, a phenomenon primarily attributed to certain advantages of low‐molecular‐weight polysaccharides, such as smaller molecular size, reduced steric hindrance, enhanced binding capacity to active sites, and improved solubility and bioavailability [42]. In the present study, the Mw of ATP was 159.1 kDa, which may confer potential anti‐aging and antioxidative activities. Although monosaccharide composition and Mw do not constitute the exclusive determinants of ATP activity, they provide a reasonable explanation at the material basis level for the anti‐liver‐aging effects of ATP observed in subsequent experiments, and also suggest that its activity may arise from the synergistic interplay of specific monosaccharide ratios and Mw range, rather than being dictated by a single structural factor alone.

To predict the mechanisms of action of ATP in treating liver aging, this study employed a combined bioinformatics and network pharmacology analytical strategy to explore the potential targets of ATP intervention in liver aging. GO and pathway enrichment analyses revealed that the targets of ATP were predominantly involved in inflammatory response‐ and oxidative stress‐related pathways, which is consistent with the key pathological mechanisms of liver aging uncovered by GSEA. It has been established that oxidative stress and chronic inflammation are prominent hallmarks of aging, participating in the aging process and closely associated with the development and progression of aging‐related diseases [43, 44]. Moreover, oxidative stress can serve as a critical trigger for the activation of inflammatory responses in liver tissue, leading to the overexpression and secretion of pro‐inflammatory cytokines [45], while chronic inflammatory responses not only induce tissue damage in liver tissue but also further exacerbate oxidative stress [46]. Previous studies have demonstrated that naturally derived compounds such as Dendrobium officinale polysaccharides and *Arctium lappa L*. polysaccharide (ALP) can ameliorate D‐gal‐induced liver aging by enhancing antioxidative and anti‐inflammatory activities [14, 47]. It has also been reported that ATP possesses anti‐inflammatory effects [48], which corroborates the reliability of the network pharmacology analysis results in this study. To further predict the potential molecular mechanisms underlying ATP‐mediated regulation of liver aging, this study integrated inflammation‐ and oxidative stress‐related gene sets from the GeneCards database and identified 63 relevant potential targets. Subsequently, three core targets, namely TLR4, NFE2L2, and CASP1, were predicted through WGCNA analysis and machine learning. Of note, although CASP1 was also predicted as a core target, given the pivotal roles of TLR4 and NFE2L2 in regulating inflammation and oxidative stress, respectively, this study prioritized experimental validation of these two targets and their associated pathways. The specific differences in their regulatory mechanisms remain to be addressed in future investigations.

The experimental results of this study validated the above predictions. Histopathological examination revealed that ATP significantly ameliorated D‐gal‐induced aging‐related pathological changes in liver tissue, attenuated morphological structural damage to hepatocytes, and reduced inflammatory cell infiltration. Immunohistochemistry further confirmed that ATP treatment significantly downregulated the expression levels of TLR4 and its downstream NF‐κB in aged liver tissue. Moreover, molecular docking demonstrated that all six monosaccharide components of ATP could stably bind to TLR4. This finding suggests that TLR4 may serve as a direct potential target of ATP in exerting its anti‐inflammatory aging effects, and that the hepatoprotective effect of ATP is closely associated with its inhibition of TLR4/NF‐κB‐mediated inflammatory responses. As a member of the Toll‐like receptor family, TLR4 can be activated by various damage‐associated molecular patterns, subsequently initiating NF‐κB‐mediated pro‐inflammatory cytokine responses through the MyD88‐dependent pathway, a mechanism that has been established as one of the core driving factors of inflammaging [49]. Studies have shown that polysaccharides from Cimicifuga heracleifolia and Ilex asprella can ameliorate liver injury and hepatic inflammation, respectively, through inhibition of the TLR4/NF‐κB pathway [50, 51], suggesting that TLR4/NF‐κB may serve as a common signaling hub through which multiple plant‐derived polysaccharides exert their protective effects in the liver. Of note, Zhong et al. previously reported that ATP could alleviate neuroinflammation by inhibiting TLR4/NF‐κB [21]. The pro‐inflammatory role of the TLR4/NF‐κB pathway in hepatic inflammation has also been extensively documented [52, 53]. However, whether ATP can ameliorate liver aging through the TLR4/NF‐κB pathway has not yet been reported. In this study, through a combination of bioinformatics prediction and in vivo experimental validation, we for the first time linked the anti‐liver‐aging effect of ATP to the TLR4/NF‐κB pathway. It should be noted that the present study still lacks pathway‐specific knockdown or inhibition experiments, and thus the causal role of this pathway remains to be confirmed in future investigations.

Unlike the linear inhibition of inflammatory pathways, ATP exhibited a more complex pattern in regulating oxidative stress. Although the Nrf2 signaling pathway may be activated by oxidative stress in aged livers [54], the present study observed a discordant expression trend between NFE2L2 and Nrf2 protein levels. GEO data analysis revealed upregulation of NFE2L2 expression, whereas immunohistochemical results showed a significant decrease in the number of Nrf2‐positive cells in the liver aging model. One possible explanation is that the high transcriptional activity of the NFE2L2 gene represents a compensatory response to oxidative stress, as studies by Rushing et al. have indicated that transcription of the NFE2L2 gene encoding Nrf2 increases during oxidative stress [55]; however, due to translational suppression mediated by mechanisms such as miR‐146a, the newly synthesized protein fails to accumulate effectively, ultimately resulting in decreased Nrf2 protein levels [56]. Furthermore, Nrf2 can directly regulate numerous genes that act as negative feedback inhibitors, capable of suppressing cytokine production and NF‐κB‐mediated inflammation [57]. This is consistent with the concomitant downregulation of TLR4/NF‐κB and upregulation of Nrf2 observed in this study, suggesting that ATP may delay liver aging through synergistic anti‐inflammatory and antioxidative mechanisms, rather than acting in isolation on a single pathway. It should be noted that the current evidence is primarily derived from histopathological and immunohistochemical analyses, and quantitative validation of downstream key effector molecules such as TNF‐α, iNOS, and COX‐2 at the protein (Western blot), transcriptional (qPCR), and secretion (ELISA) levels has not yet been performed. Therefore, the existing conclusions are predominantly correlative in nature, and causal evidence remains to be supplemented in future studies.

An interesting finding of this study was that although ATP significantly upregulated Nrf2 expression, it concurrently suppressed the aberrant elevation of HO‐1. Traditionally, HO‐1, as a key antioxidant enzyme downstream of Nrf2, is generally considered to exhibit expression positively correlated with Nrf2 activity [58]. In this regard, the present study performed validation through immune infiltration analysis and single‐cell sequencing, revealing that the elevation of HO‐1 in aged livers may not be primarily driven by enhanced Nrf2 transcriptional activity, but rather is closely associated with a significant increase in Kupffer cell numbers and their activated status. The immunohistochemical results of this study also supported this conclusion—HO‐1 was predominantly expressed in Kupffer cells. This finding is highly consistent with the literature discussing that HO‐1 expression in aged livers originates from Kupffer cells [59], namely that the upregulation of HO‐1 during the aging process is largely attributable to increased macrophage abundance. On this basis, the present study proposes that the inhibitory effect of ATP on HO‐1 actually reflects its negative regulation of aberrant Kupffer cell activation, rather than a simple antagonism of Nrf2 function. Studies have shown that under chronic inflammatory or oxidative stress conditions, sustained high expression of HO‐1 can lead to iron accumulation, mitochondrial dysfunction, and maintenance of a pro‐inflammatory microenvironment [60, 61], thereby exacerbating liver tissue injury. This suggests that ATP‐mediated suppression of HO‐1 may be indirectly achieved, in part, through attenuation of Kupffer cell inflammation. This finding indicates that when evaluating the antioxidative effects of the Nrf2 pathway, the cellular source of HO‐1 expression and its pathophysiological context should be taken into consideration, and also provides a new perspective for understanding the cell‐specific actions of natural polysaccharides in liver aging.

## 5 Conclusions

In summary, this study integrated structural characterization, network pharmacology, molecular docking, and in vivo validation to systematically investigate the role of ATP in modulating liver aging and its potential mechanisms. The results indicate that ATP can delay liver aging, and its effects are closely associated with the regulation of inflammatory responses and oxidative stress mediated by the TLR4/NF‐κB and Nrf2 signaling pathways. In addition, ATP may modulate excessive activation of HO‐1, thereby contributing to its protective effects against liver aging. These findings provide new insights and preliminary evidence for a deeper understanding of the mechanism by which ATP combats liver aging.

### 5.1 Limitations of the Study

The following limitations of this study should be acknowledged:

The crude total polysaccharides reflect the multi‐component synergistic characteristics of natural products; however, their pharmacological effects cannot be attributed to any specific polysaccharide chain or monosaccharide moiety. The structure–activity relationship elucidation of ATP and identification of its key active structural domains still require further clarification through isolation, purification, and structural characterization (e.g., nuclear magnetic resonance). Furthermore, target screening in this study was based on publicly available human datasets, and in vivo validation was conducted using a D‐gal‐induced rat aging model. This model is widely used for the preliminary evaluation of the efficacy of anti‐aging compounds due to its practicality and reproducibility [62]. Although it effectively simulates core aging‐related phenotypes such as oxidative stress, chronic inflammation, and tissue degeneration, limitations inherent to the rodent species and the modeling approach mean that it still differs from natural physiological aging in humans in aspects such as basal metabolic rate and telomere maintenance mechanisms [63]. Further studies are needed to observe trends in relevant targets using human tissue samples or clinical data to provide a reference basis for translational applications.

Notwithstanding the aforementioned limitations, the present study still provides a valuable foundation for future research on ATP in delaying liver aging and for the elucidation of its underlying mechanisms.

## Author Contributions

**Yikuan Du**: conceptualization, data curation, formal analysis, Writing – original draft. **Jiahui Li**: conceptualization, data curation, formal analysis, writing – original draft. **Yuting Lin**: investigation, visualization. **Jiawen Luo**: investigation, visualization. **Weien Zhang**: validation, methodology. **Feng Chen**: methodology, investigation. **Zhonghan Liang**: methodology. **Jiaxin Zhu**: project administration. **Jiaqi Li**: project administration. **Chenyue Yuan**: supervision. **Yidian Liu**: resources. **Xuan Liu**: software. **Yang Zhang**: supervision. **Qiaohong Zhong**: writing – review & editing. **Yanling Jiang**: software. **Songsong Yang**: validation. **Chunling Ma**: writing – review & editing. **Zhi Chen**: writing – review & editing. **Chun Yang**: funding acquisition. All authors contributed to the article and approved the submitted version.

## Funding

This research was funded in part by a grant from the Guangdong Basic and Applied Basic Research Foundation (No. 2025A1515012615), 2025 Guangdong Medical University Clinical–Basic Science Innovation Special Program (Nos. GDMULCJC2025223, GDMULCJC2025229), the Guangdong Medical University Students' Innovation and Entrepreneurship Training Program (Nos. GDMU2024088, GDMU2024114, GDMU2024130, GDMU2024193, GDMU2024232, GDMU2025006, GDMU2025127, GDMU2025174, GDMU2025218), the Provincial and National College Students' Innovation and Entrepreneurship Training Program (Nos. S202510571092, 202610571006), Guangdong Provincial Science and Technology Innovation Strategy Special Project (College Students' Science and Technology Innovation Cultivation Project) (pdjh2025bk103), Guangdong Medical University Undergraduate Innovation and Entrepreneurship Education Base Project (JDXM2024136, JDXM2024158, JDXM2024183, JDXM2024190, JDXM2024071F, JDXM2025034, JDXM2025083, JDXM2025115, JDXM2025190).

## Conflicts of Interest

The authors declare no conflicts of interest.

## Data Availability

Data will be made available on request.
